# HIV and Hepatitis C Virus Coinfection, Cameroon

**DOI:** 10.3201/eid1303.061069

**Published:** 2007-03

**Authors:** Christian Laurent, Anke Bourgeois, Mireille Mpoudi, Christelle Butel, Eitel Mpoudi-Ngolé, Eric Delaporte

**Affiliations:** * Institut de Recherche pour le Développement/University of Montpellier 1, Montpellier, France; † Military Hospital, Yaoundé, Cameroon

**Keywords:** HIV, hepatitis, coinfection, epidemiology, prevalence, Africa, letter

**To the Editor:** Coinfection with HIV and hepatitis C virus (HCV) is now a major public health concern worldwide, owing both to its high prevalence (4–5 million persons of 40 million infected by HIV) and to interactions between the 2 diseases in terms of their diagnosis, natural course, and treatment (*1*,*2*). Although Africa is the continent by far the most badly affected by both HIV and HCV infections, data on coinfection in the general population are lacking. In Cameroon, a central African country, the HCV seroprevalence is among the highest in the world (13.8%) (*3*). We have also reported a high seroprevalence of HIV in a general population of southern Cameroon (7.4%), and especially in young women (22.5%) (*4*). Here, we investigated the prevalence of HIV/HCV coinfection in this population.

A population-based, cross-sectional survey was conducted in September 2001 in 3 villages of the East Province of Cameroon (250 km from Yaoundé, the capital city). The study methods, the baseline characteristics of the participants, and the HIV seroprevalence have been described in detail elsewhere (*4*). Briefly, all inhabitants >15 years of age were eligible for the survey. After giving their informed consent, the participants were interviewed by using a standard verbal questionnaire, in French or in a local language, during door-to-door visits. Blood samples were collected by peripheral venipuncture, and serum was screened for antibodies to HCV by using an enzyme immunoassay (INNOTEST HCV Ab IV, Innogenetics, Ghent, Belgium). Samples with indeterminate results were retested. All positive and twice-indeterminate samples were confirmed with a third-generation line immunoassay (INNO-LIA HCV Ab III update, Innogenetics). Serologic screening for HIV infection was based on an enzyme immunoassay (Murex HIV-1.2.O, Abbott, Rungis, France). All positive samples were confirmed by using a line immunoassay (INNO-LIA HIV-1+2, Innogenetics).

Among the 484 participants, 256 were women (52.9%), and the median age was 34 years (interquartile range 23–52 years). Most participants (93.6%) were Bantus; the remainder were pygmies. Seven persons refused venipuncture after the interview, and 1 sample could not be analyzed. These 8 persons did not differ from the rest of the study population in term of sex (50.0% women vs. 47.1% women), but they were slightly younger (median, 26.8 years vs. 34.9 years). Of the 476 available samples, respectively 19 (4.0%) had indeterminate HCV serologic results, and 5 (1.1%) had indeterminate HIV serologic results. The overall seroprevalence rates were 21.0% (95% confidence interval [CI] 17.4%–24.9%) for HCV and 7.4% (95% CI 5.2%–10.1%) for HIV. Only 3 patients (0.6%) had positive results for both infections: a man 29 years of age and 2 women ages 36 and 52 years.

The Figure shows the seroprevalence rates of HCV and HIV according to sex and age. Multivariate random-effects logistic regression analyses showed different risk factors for the 2 infections. The HCV seroprevalence was associated with age (<45 vs. ≥45 years, odds ratio [OR] 13.04; 95% CI 6.73–25.30; p<0.001), sex (men vs. women, OR 2.02; 95% CI 1.17–3.47; p = 0.01) and the ethnic group (Bantus vs. pygmies, OR 10.98; 95% CI 1.31–92.42; p = 0.03). In contrast, the HIV seroprevalence was higher in women than in men (OR 10.22; 95% CI 3.19–32.80; p<0.001). No specific risk factors were found in men, whereas women who were unmarried (OR 6.49; CI 2.45–17.17; p<0.001) or school-educated (OR 7.12; 95% CI 1.59–31.78; p = 0.01), or those with a history of sexually transmitted infections (OR 2.92; 95% CI 1.08–7.89; p = 0.03) had higher rates than other women.

**Figure:**
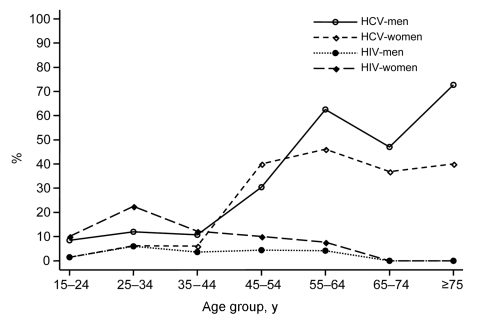
Seroprevalence rates of hepatitis C virus (HCV) and HIV infection by sex and age in the general population of southern Cameroon, 2001.

HIV/HCV coinfection is therefore rare in this general population, which lives in an area where both HCV and HIV are endemic. This finding could be related to the dissimilar epidemiologic patterns of the 2 infections. Indeed, HIV infection mainly affects young persons, especially young women, while HCV infection is more frequent in older persons of both sexes. We have previously postulated that HIV is likely to be transmitted by the sexual route, in a context of commercial logging and the extensive and complex sexual networks it induces (*4*). In contrast, the route of HCV transmission is unclear. HCV seropositivity was not associated with a history of blood transfusion, injections, surgery, scarification, or tattooing. Intravenous drug use was not investigated in our study but was likely to be infrequent. Although sexual transmission could not be ruled out, especially between regular partners, the shape of the seroprevalence curves and the lack of association with HIV infection, syphilis, or other sexually transmitted infections suggests that this mode of transmission is inefficient, in keeping with other reports (*5*,*6*). Our seroprevalence curves and the study location are consistent with the hypothesis that frequent iatrogenic transmission occurred during mass medical campaigns conducted before 1960 (*7*). The rate of HCV coinfection among the HIV-infected subjects in our study (8.6%) is much lower than the overall rate (25%–30%) in North America and Europe (*1*,*2*), where intravenous drug use is a major risk factor for both infections (*8*,*9*). This rate was even in the lower range of values found among HIV-infected heterosexual persons in industrialized countries (9%–27%) (*2*). Our results therefore suggest that the high seroprevalence rates of HIV and HCV in Africa will not necessarily result in a high prevalence of HIV/HCV coinfection.

## References

[1] Rockstroh JK, Spengler U. HIV and hepatitis C virus co-infection. Lancet Infect Dis. 2004;4:437–44. 10.1016/S1473-3099(04)01059-X 15219554

[2] Alter MJ. Epidemiology of viral hepatitis and HIV co-infection. J Hepatol. 2006;44(Suppl):S6–9. 10.1016/j.jhep.2005.11.004 16352363

[3] Madhava V, Burgess C, Drucker E. Epidemiology of chronic hepatitis C virus infection in sub-Saharan Africa. Lancet Infect Dis. 2002;2:293–302. 10.1016/S1473-3099(02)00264-5 12062995

[4] Laurent C, Bourgeois A, Mpoudi M, Butel C, Peeters M, Mpoudi-Ngolé E, Commercial logging and HIV epidemic, rural Equatorial Africa. Emerg Infect Dis. 2004;10:1953–6. 15550206 10.3201/eid1011.040180 PMC3328988

[5] Laurent C, Henzel D, Mulanga-Kabeya C, Maertens G, Larouzé B, Delaporte E. Seroepidemiological survey of hepatitis C virus among commercial sex workers and pregnant women in Kinshasa, Democratic Republic of Congo. Int J Epidemiol. 2001;30:872–7. 10.1093/ije/30.4.872 11511619

[6] Flamm SL. Chronic hepatitis C virus infection. JAMA. 2003;289:2413–7. 10.1001/jama.289.18.2413 12746366

[7] Nerrienet E, Pouillot R, Lachenal G, Njouom R, Mfoupouendoun J, Bilong C, Hepatitis C virus infection in Cameroon: a cohort-effect. J Med Virol. 2005;76:208–14. 10.1002/jmv.20343 15834878

[8] Hagan H, Thiede H, Des Jarlais DC. HIV/hepatitis C virus co-infection in drug users: risk behavior and prevention. AIDS. 2005;19(suppl 3):S199–207. 10.1097/01.aids.0000192090.61753.d4 16251818

[9] Mohsen AH, Murad S, Easterbrook PJ. Prevalence of hepatitis C in an ethnically diverse HIV-1-infected cohort in south London. HIV Med. 2005;6:206–15. 10.1111/j.1468-1293.2005.00291.x 15876288

